# Supplementary material for: Characterizing polarization in online vaccine discourse—A large-scale study
Source: PLoS One. 2022 Feb 9;17(2):e0263746. doi: 10.1371/journal.pone.0263746 (PMC8827439; doi:10.1371/journal.pone.0263746)
Supplement: S1 Appendix — More details on uncertainties and robustness to sentiment thresholds is provided in S1 Appendix. (PDF) [file pone.0263746.s001.pdf]

S1 Appendix: Uncertainties and robustness.

This supplementary information provides some details regarding uncertainties in the figures presented in the main paper, as well as robustness analyses. The latter recreate key figures from the main paper while varying the sentiment threshold used to assign user stance, effectively demanding that users post tweets expressing increasingly strong sentiments regarding human vaccination to be regarded as anti- or pro-vaccine. Section 1 provides details on uncertainty analysis, Section 2 investigates robustness to the sentiment threshold, and Section 3 summarizes the data loss associated with tightening the definitions of user stance by increasing the sentiment threshold.

1 Uncertainty analysis

Figures 1 to 3 reproduce the figures from the main paper whose error bars are hard to process visually, due to their small sizes. For convenience, Tables 1 to 3 provide the exact data, including error bars, that underlie the plots.

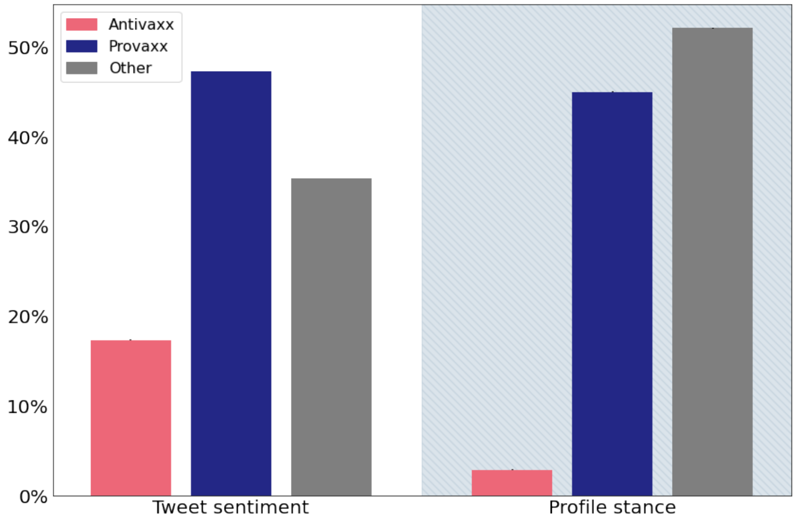

Figure 1: Map showing the ratio of the estimated number of tweets expressing anti- and provaccine sentiment in the United States. Darker colors indicate a larger fraction of antivaxx tweets.

	Tweet sentiment	User stance
% AV	17.4	2.9
Error AV	0.0145	0.0139
% PV	47.3	45.0
Error PV	0.0185	0.041
% Other	35.3	52.1
Error Other	0.0179	0.041

Table 1: Overview of the estimated distribution over tweet sentiments and user stances shown in Fig. 1, as well as the associated errors. For tweet sentiments, the error is given as $\sqrt{\langle\sigma_i^2\rangle/N}$, where σ_i is given by $p_i(1-p_i)$, where p_i is the probability of tweet i expressing the sentiment in question. For user stance, the error is given as $\sqrt{f_i(1-f_i)/N}$, where f_i if the fraction of users labeled with the given stance, and N is the number of users.

URL	% AV	Error AV	% PV	Error PV
youtube.com	22.5	0.142	1.44	0.0107
naturalnews.com	10.5	0.105	0.165	0.00366
facebook.com	2.09	0.0488	1.7	0.0117
healthimpactnews.com	2.08	0.0487	0.064	0.00228
newspunch.com	2.04	0.0482	0.0463	0.00194
worldtruth.tv	1.96	0.0473	0.0188	0.00124
vaccineimpact.com	1.8	0.0453	0.0249	0.00142
instagram.com	1.51	0.0417	0.397	0.00567
foxnews.com	1.37	0.0397	0.594	0.00693
newstarget.com	1.14	0.0363	0.00675	0.00074
bbc.co.uk	0.0745	0.00931	2.44	0.0139
nytimes.com	0.154	0.0134	2.02	0.0127
cdc.gov	0.495	0.024	1.47	0.0108
edition.cnn.com	0.485	0.0237	1.38	0.0105
reuters.com	0.0443	0.00718	1.2	0.00982
theguardian.com	0.148	0.0131	1.16	0.00965
bioportfolio.com	0	0	1.14	0.00956
sciencedaily.com	0.0233	0.00521	1.14	0.00955

Table 2: Estimated percentages of links of strongly pro- and anti-vaccine profiles which point to selected domains. Error bars are calculated as $\sqrt{f_i(1-f_i)/N}$, where N is the number of tweets form users of the given stance and f_i is the fraction of such tweets pointing to domain i.

Stance	Link category	Tweets in category (%)	Error (%)
PV	pseudoscience	0.0728	6.48e-05
AV	pseudoscience	2.64	0.00859
All	pseudoscience	0.399	0.000244
PV	social	2.82	0.00244
AV	social	3.52	0.0113
All	social	5.14	0.00299
PV	science	5.17	0.00437
AV	science	0.572	0.0019
All	science	2.91	0.00174
PV	conspiracy	0.0735	6.54e-05
AV	conspiracy	6.43	0.0201
All	conspiracy	0.747	0.000456
PV	news	16.6	0.0124
AV	news	3.18	0.0103
All	news	10.6	0.00582
PV	commercial	0.445	0.000395
AV	commercial	18.5	0.0504
All	commercial	4.94	0.00289
PV	youtube	1.41	0.00123
AV	youtube	21.5	0.0564
All	youtube	5.03	0.00293

Table 3: Exact data underlying the link category analysis depicted in Fig. 3. Error bars are calculated as $\sqrt{f_i(1-f_i)/N}$, where f_i is the fraction of links shared by users of the given stance which point to domains in category i, and N is the total number of links found by users of that stance.

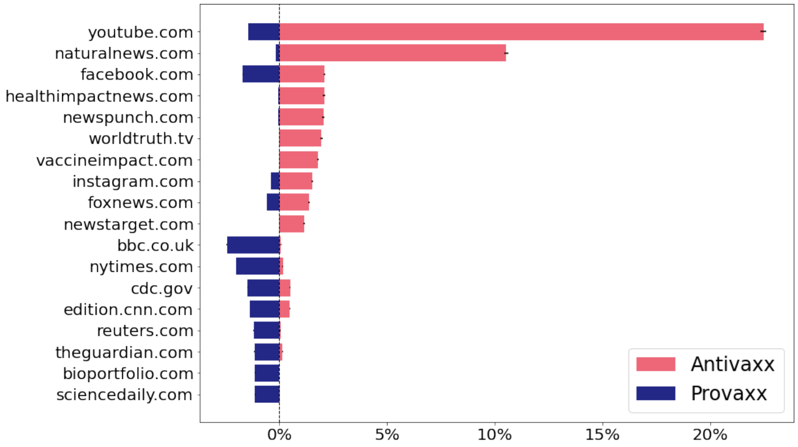

Figure 2: The top 10 most linked to domains by strongly antivaxx and provaxx profiles, including error bars. The exact percentages and errors are provided in Table 2.

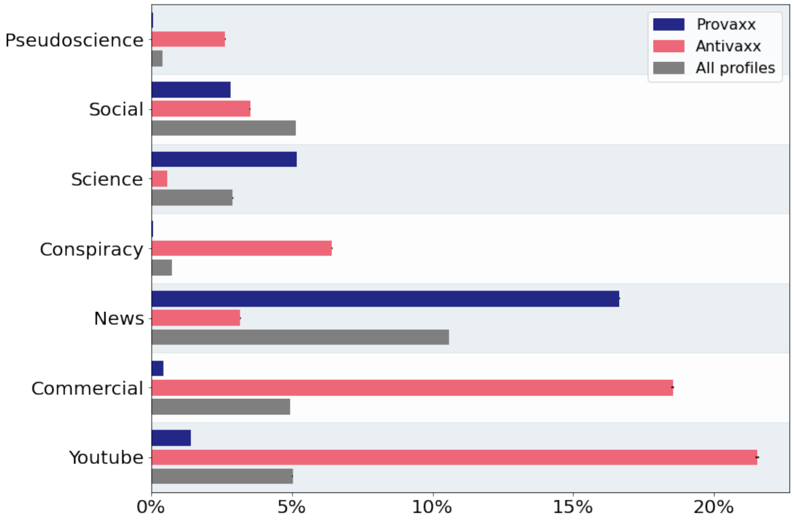

Figure 3: A similar analysis as that shown in Fig. 2, but breaking links down by category, as described in the main paper, and comparing domains linked to by users with strong stances, with the overall population of Dataset 2, i.e. in tweets discovered by querying for vaccination-related keywords. Error bars are present, but hard to process visually, so exact values are provided in Table 3.

2 Robustness analysis

This section investigates what happens when the somewhat arbitrary criteria for assigning user stance in the main paper are varied. Specifically, we vary the threshold for tweet vaccine sentiment which 50% of a user's tweets must be above in order to assign stance. In effect, this places more strict demands on when users are assigned a stance of pro- or anti-vaccine. For example, increasing the threshold to 90%, will place the constraint that in order to be considered pro-/anti-vaccine, our machine learning classifier must assign over 50% of a user's tweets a probability of at least 90% of expressing pro-/anti-vaccine sentiment.

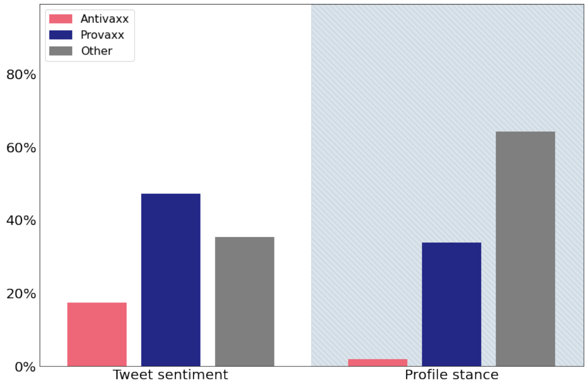

Figure 4: **Distribution of tweet sentiments and user stances**. Users were labeled as each stance only if at least 50% of the user's tweets were assigned probabilities of at least 60% of expressing the corresponding sentiment.

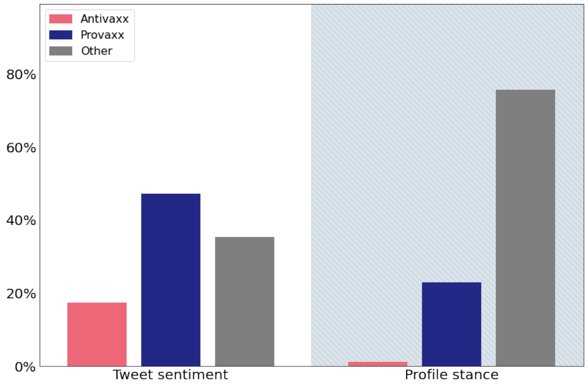

Figure 5: **Distribution of tweet sentiments and user stances**. Users were labeled as each stance only if at least 50% of the user's tweets were assigned probabilities of at least 70% of expressing the corresponding sentiment.

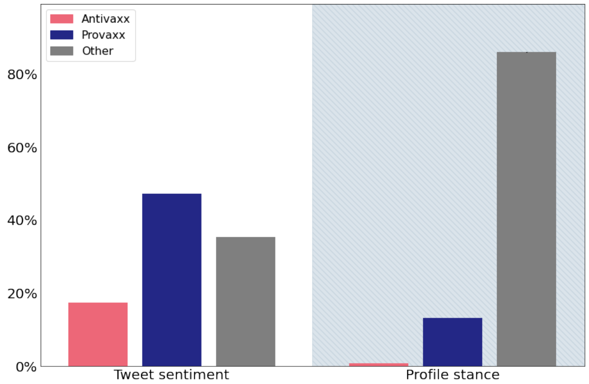

Figure 6: **Distribution of tweet sentiments and user stances**. Users were labeled as each stance only if at least 50% of the user's tweets were assigned probabilities of at least 80% of expressing the corresponding sentiment.

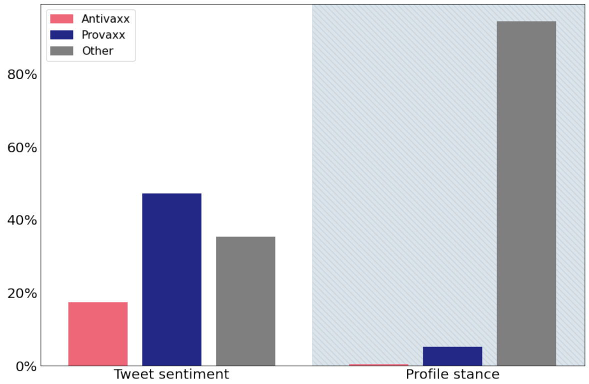

Figure 7: **Distribution of tweet sentiments and user stances**. Users were labeled as each stance only if at least 50% of the user's tweets were assigned probabilities of at least 90% of expressing the corresponding sentiment.

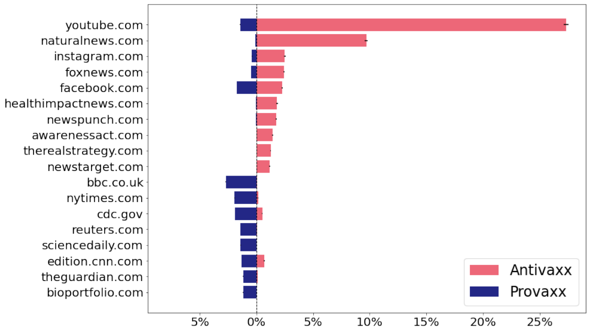

Figure 8: **Sources for URLs posted by users with strongly expressed vaccine stances**. Users were labeled as each stance only if at least 50% of the user's tweets were assigned probabilities of at least 60% of expressing the corresponding sentiment.

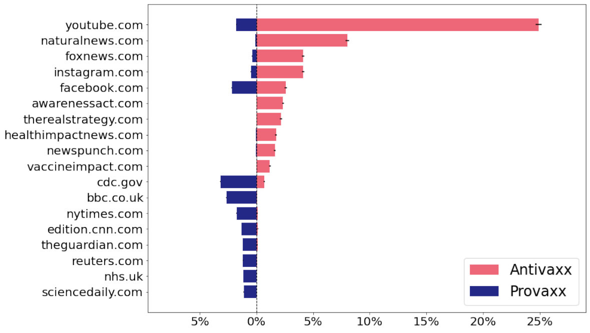

Figure 9: **Sources for URLs posted by users with strongly expressed vaccine stances**. Users were labeled as each stance only if at least 50% of the user's tweets were assigned probabilities of at least 70% of expressing the corresponding sentiment.

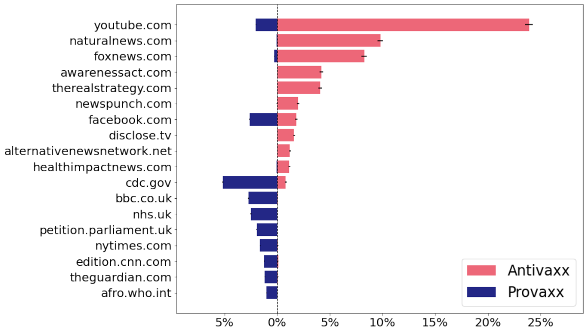

Figure 10: **Sources for URLs posted by users with strongly expressed vaccine stances**. Users were labeled as each stance only if at least 50% of the user's tweets were assigned probabilities of at least 80% of expressing the corresponding sentiment.

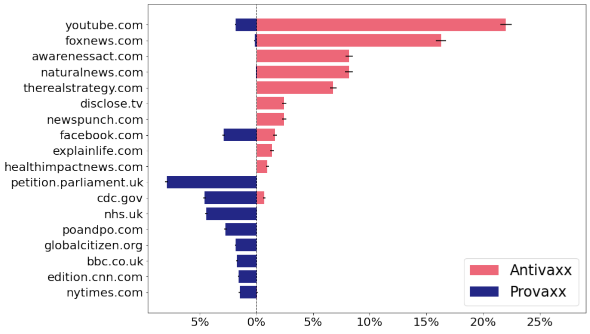

Figure 11: **Sources for URLs posted by users with strongly expressed vaccine stances**. Users were labeled as each stance only if at least 50% of the user's tweets were assigned probabilities of at least 90% of expressing the corresponding sentiment.

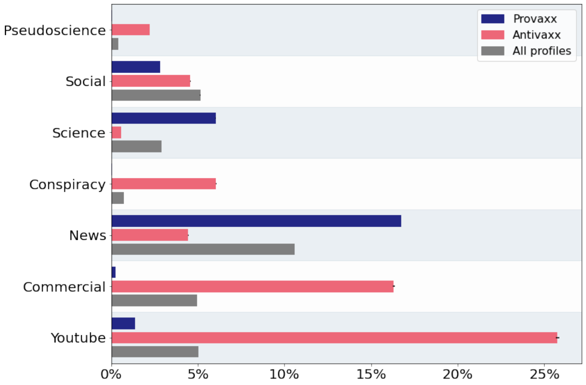

Figure 12: **Interplay between vaccine stance and sources by URL category**. Users were labeled as each stance only if at least 50% of the user's tweets were assigned probabilities of at least 60% of expressing the corresponding sentiment.

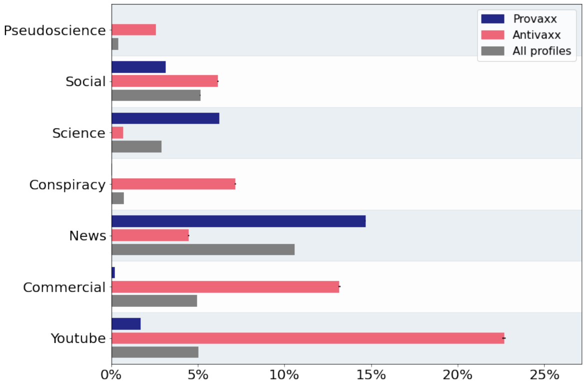

Figure 13: **Interplay between vaccine stance and sources by URL category**. Users were labeled as each stance only if at least 50% of the user's tweets were assigned probabilities of at least 70% of expressing the corresponding sentiment.

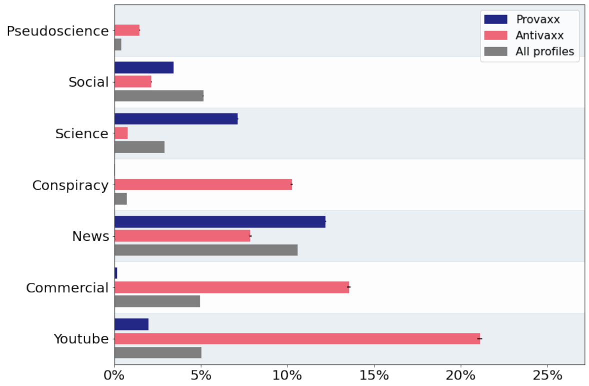

Figure 14: **Interplay between vaccine stance and sources by URL category**. Users were labeled as each stance only if at least 50% of the user's tweets were assigned probabilities of at least 80% of expressing the corresponding sentiment.

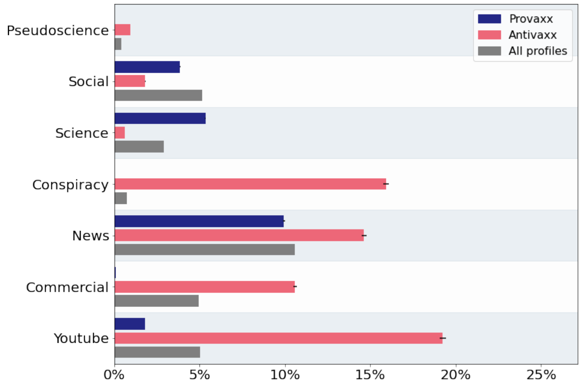

Figure 15: **Interplay between vaccine stance and sources by URL category**. Users were labeled as each stance only if at least 50% of the user's tweets were assigned probabilities of at least 90% of expressing the corresponding sentiment.

3 Data retention

This section summarizes the data loss associated with tightening the restrictions on which profiles are viewed as having an anti- or pro-vaccine stance in the analyses. Table 4 shows the number of profiles associated with each stance for each of the sentiment thresholds used in the robustness analyses presented here.

Sentiment threshold	AV	PV
50%	43,071	663,026
60%	28,541	498,140
70%	18,622	338,300
80%	10,846	195,715
90%	5,034	76,826

Table 4: When increasingly strict conditions are used to label profile stances as anti- or pro-vaccine, fewer profiles get assigned such labels. Increasing the sentiment threshold from 50% to 90% reduces the number of profiles considered anti-vaccine from $\sim 43,000$ to $\sim 5,000$. This amounts to considering profiles expressing increasingly strong anti-vaccine sentiments, and accounts for the differences among the figures in Section 2. Profiles not assigned a stance under the given criteria are retained as a reference group in some of the figuresm in which case the total number of profiles is constant at 1,473,815.
