# Supplementary material for: Characterizing polarization in online vaccine discourse—A large-scale study
Source: PLoS One. 2022 Feb 9;17(2):e0263746. doi: 10.1371/journal.pone.0263746 (PMC8827439; doi:10.1371/journal.pone.0263746)
Supplement: S2 Appendix — A short analysis of tweet sentiment by American state, of potential relevancy to researchers interested in the interplay between state policy/regulations and Twitter discourse, is presented in S2 Appendix. (PDF) [file pone.0263746.s002.pdf]

S2 Appendix: Geographical analysis of tweets originating from the USA.

The present appendix presents a visualization of the vaccine sentiment expressed in tweets originating from the USA, broken down at the state level. This is shown in Fig. 1. The raw data used for this plot are presented in Table 1. As very few tweets were encoded with GPS-coordinates, we relied on a large dataset (Dataset 1 in the main paper) to infer location for a number of tweets. This procedure is outlined below.

In order to assess the location from which tweets were sent, we used a three-level approach to determine locations. A small fraction (.2%) of tweets were posted with GPS coordinates in their metadata. When GPS coordinates were available, tweet location could be inferred directly from the latitude/longitude values.

When GPS coordinates were not available, we checked the tweet metadata for a *location string*, i.e. a text string with which the user describes their current location, such as the string 'New York City'. When a location string was available, we were able to use the OpenStreetMaps API to unambigously assign a location in 4.2% of tweets.

In cases where the location could not be unambiguously inferred from the location string, we used a side-channel approach utilizing dataset 1. Specifically, we used a mapping of each location string of geotagged tweets in dataset 1 to the maximum likelihood distribution over US counties in which tweets containing that location string originated. From that, we computed a probability distribution over states, and assigned the most likely state to tweets based on their location strings. This approach was successful for 11.5% of tweets.

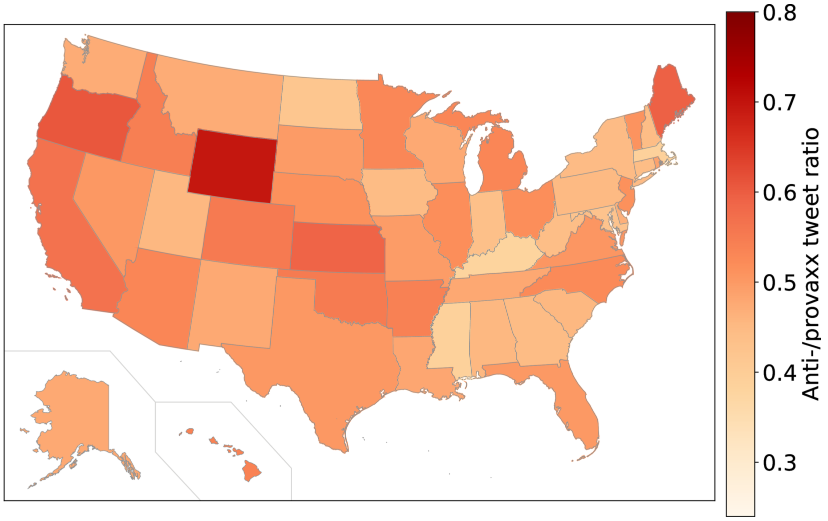

Figure 1: Map showing the ratio of the estimated number of tweets expressing anti- and provaccine sentiment in the United States. Darker colors indicate a larger fraction of antivaxx tweets. The map was produced using the open source matplotlib basemap toolkit.

Table 1: **Twitter vaccine sentiment by US state**

State	Name	N tweets	AV tweets	PV tweets	% AV	% PV	Ratio
AK	Alaska	1356	344	1012	25.4	74.6	0.340
AL	Alabama	4994	1218	3776	24.4	75.6	0.323
AR	Arkansas	566	162	404	28.6	71.4	0.401
AZ	Arizona	2874	808	2066	28.1	71.9	0.391
CA	California	17317	5223	12094	30.2	69.8	0.432
CO	Colorado	2741	805	1936	29.4	70.6	0.416
CT	Connecticut	8334	1994	6340	23.9	76.1	0.315
DC	D.C.	24039	4649	19390	19.3	80.7	0.240
DE	Delaware	7605	1901	5704	25.0	75.0	0.333
FL	Florida	66207	17564	48643	26.5	73.5	0.361
GA	Georgia	3063	735	2328	24.0	76.0	0.316
HI	Hawaii	2457	699	1758	28.4	71.6	0.398
IA	Iowa	12607	3024	9583	24.0	76.0	0.316
ID	Idaho	8535	2462	6073	28.8	71.2	0.405
IL	Illinois	38571	10600	27971	27.5	72.5	0.379
IN	Indiana	18822	4427	14395	23.5	76.5	0.308
KS	Kansas	110937	34979	75958	31.5	68.5	0.461
KY	Kentucky	37649	8141	29508	21.6	78.4	0.276
LA	Louisiana	10314	2645	7669	25.6	74.4	0.345
MA	Massachusetts	27919	6103	21816	21.9	78.1	0.280
MD	Maryland	26999	6277	20722	23.2	76.8	0.303
ME	Maine	8395	2665	5730	31.7	68.3	0.465
MI	Michigan	34337	9665	24672	28.1	71.9	0.392
MN	Minnesota	18063	5071	12992	28.1	71.9	0.390
MO	Missouri	20294	5348	14946	26.4	73.6	0.358
MS	Mississippi	19915	4359	15556	21.9	78.1	0.280
MT	Montana	25576	6446	19130	25.2	74.8	0.337
NC	North Carolina	26192	7285	18907	27.8	72.2	0.385
ND	North Dakota	2866	657	2209	22.9	77.1	0.297
NE	Nebraska	38709	10527	28182	27.2	72.8	0.374
NH	New Hampshire	3592	861	2731	24.0	76.0	0.315
NJ	New Jersey	24648	6708	17940	27.2	72.8	0.374
NM	New Mexico	11381	2893	8488	25.4	74.6	0.341
NV	Nevada	11871	3172	8699	26.7	73.3	0.365
NY	New York	114496	27631	86865	24.1	75.9	0.318
OH	Ohio	27860	7656	20204	27.5	72.5	0.379
OK	Oklahoma	29741	8710	21031	29.3	70.7	0.414
OR	Oregon	21259	6922	14337	32.6	67.4	0.483
PA	Pennsylvania	39756	9622	30134	24.2	75.8	0.319
RI	Rhode Island	4542	1176	3366	25.9	74.1	0.349
SC	South Carolina	9281	2268	7013	24.4	75.6	0.323
SD	South Dakota	14448	3826	10622	26.5	73.5	0.360
TN	Tennessee	15541	3969	11572	25.5	74.5	0.343
TX	Texas	87051	23244	63807	26.7	73.3	0.364
UT	Utah	10703	2612	8091	24.4	75.6	0.323
VA	Virginia	45197	12114	33083	26.8	73.2	0.366
VT	Vermont	16663	4497	12166	27.0	73.0	0.370
WA	Washington	32582	8210	24372	25.2	74.8	0.337
WI	Wisconsin	26158	6646	19512	25.4	74.6	0.341
WV	West Virginia	5202	1242	3960	23.9	76.1	0.314
WY	Wyoming	8318	3159	5159	38.0	62.0	0.612
