# Supplementary material for: Characterizing polarization in online vaccine discourse—A large-scale study
Source: PLoS One. 2022 Feb 9;17(2):e0263746. doi: 10.1371/journal.pone.0263746 (PMC8827439; doi:10.1371/journal.pone.0263746)
Supplement: S3 Appendix — presents illustrations of how the number of links posted to each external domain by changes over time. (PDF) [file pone.0263746.s003.pdf]

S3 Appendix: Temporal evolution of link frequencies.

The present appendix present visualizations of how the frequency of links to various external domains evolves by year. For each year, the number of links to each domain is broken down by user stance regarding vaccination, as defined in the main paper.

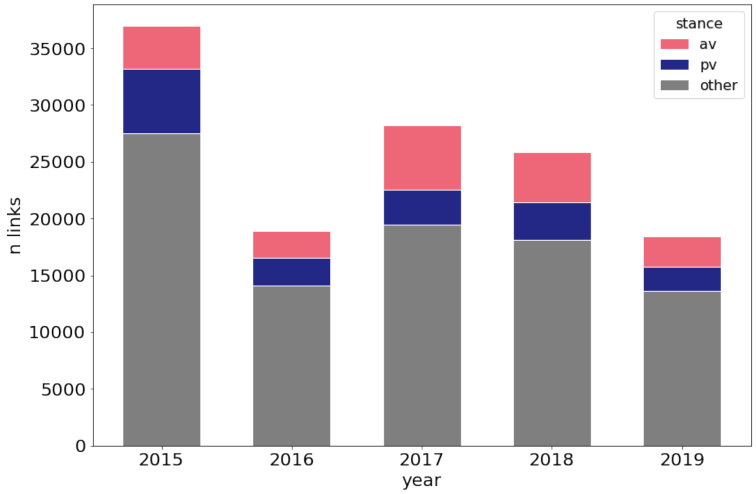

Figure 1: **youtube.com links**. Illustration of how often users of different vaccination stances post links to youtube.com over time, broken down by user stance.

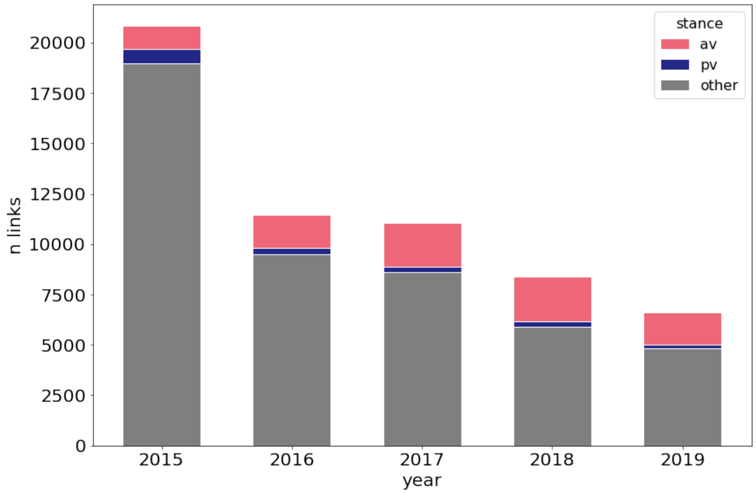

Figure 2: **naturalnews.com links**. Illustration of how often users of different vaccination stances post links to naturalnews.com over time, broken down by user stance.

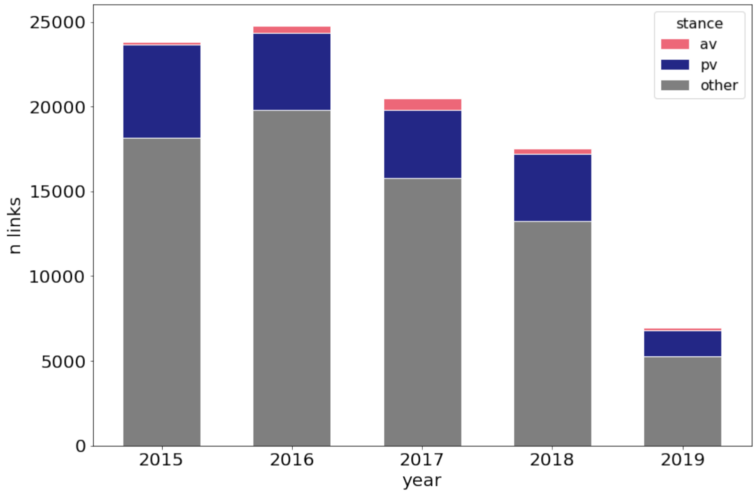

Figure 3: **facebook.com links**. Illustration of how often users of different vaccination stances post links to facebook.com over time, broken down by user stance.

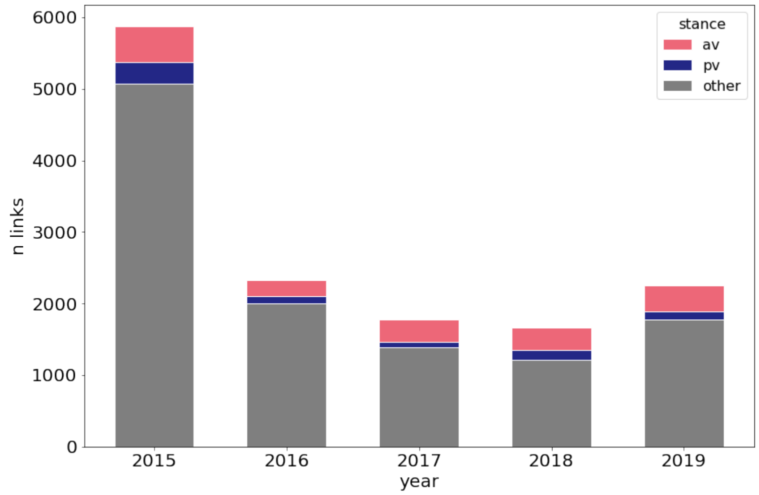

Figure 4: **healthimpactnews.com links**. Illustration of how often users of different vaccination stances post links to healthimpactnews.com over time, broken down by user stance.

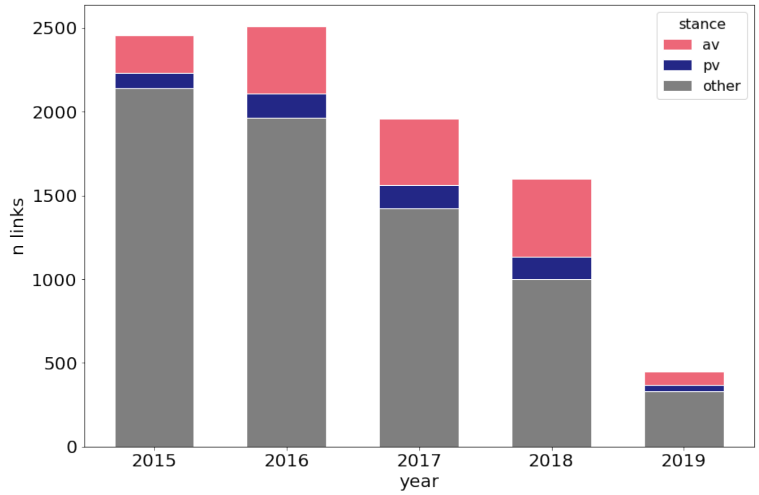

Figure 5: **newspunch.com links**. Illustration of how often users of different vaccination stances post links to newspunch.com over time, broken down by user stance.

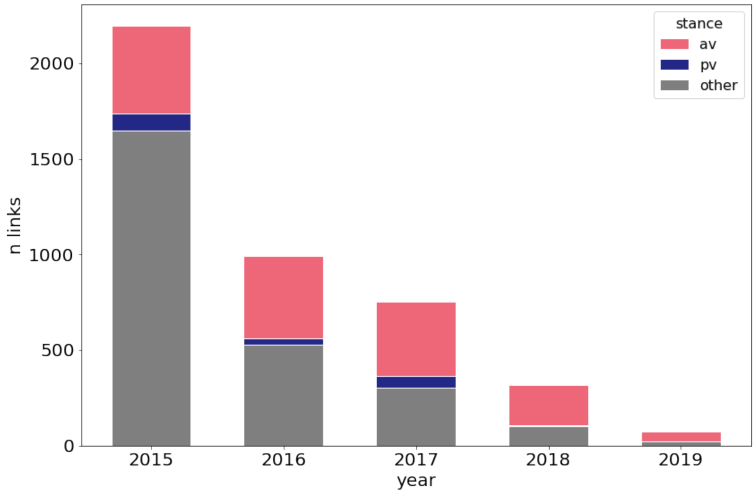

Figure 6: **worldtruth.tv links**. Illustration of how often users of different vaccination stances post links to worldtruth.tv over time, broken down by user stance.

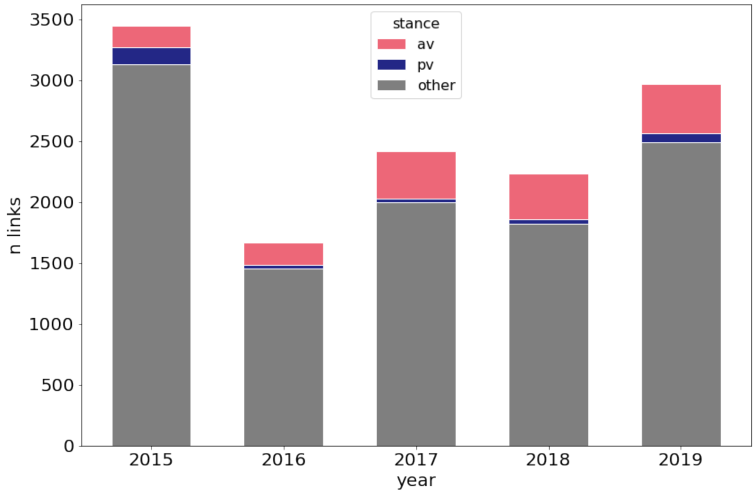

Figure 7: **vaccineimpact.com links**. Illustration of how often users of different vaccination stances post links to vaccineimpact.com over time, broken down by user stance.

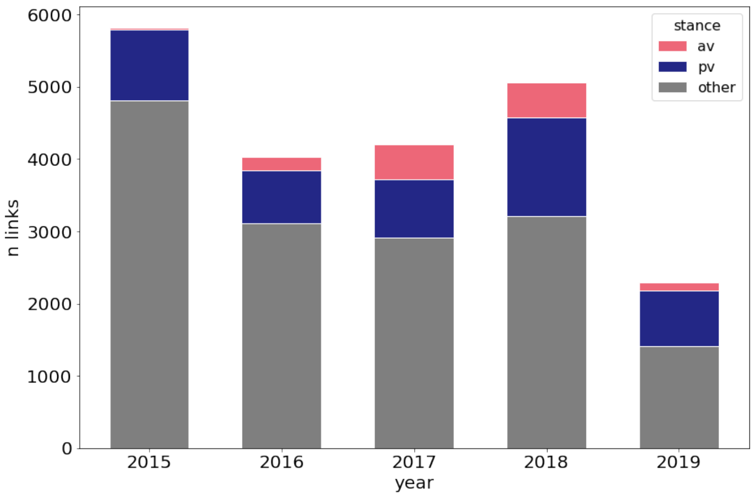

Figure 8: **instagram.com links**. Illustration of how often users of different vaccination stances post links to instagram.com over time, broken down by user stance.

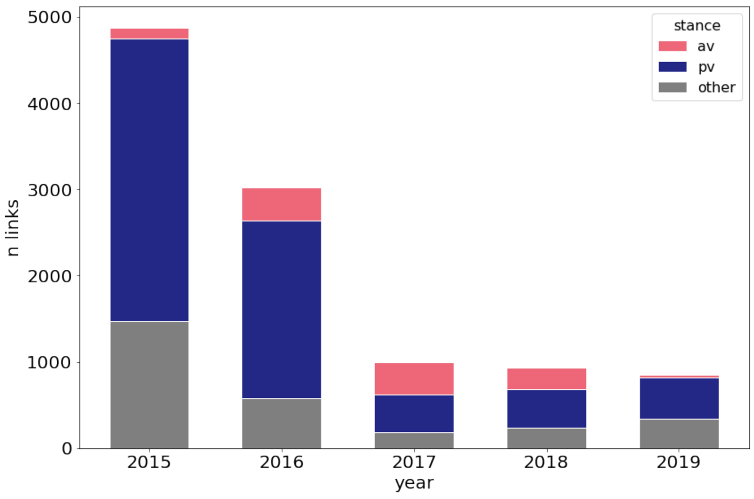

Figure 9: **foxnews.com links**. Illustration of how often users of different vaccination stances post links to foxnews.com over time, broken down by user stance.

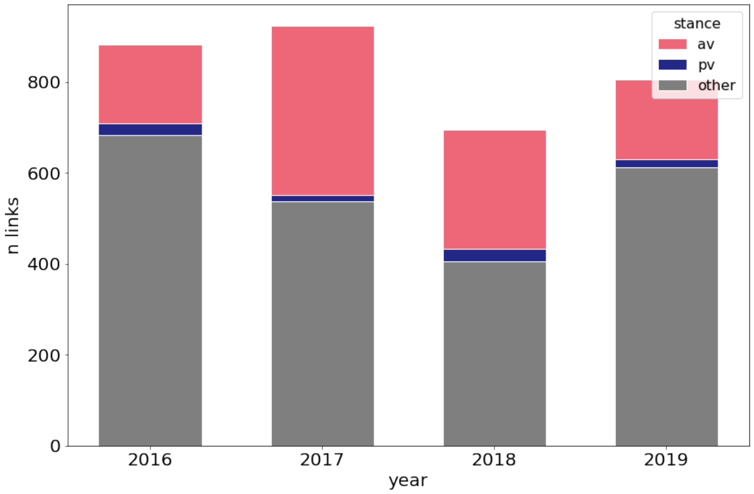

Figure 10: **newstarget.com links**. Illustration of how often users of different vaccination stances post links to newstarget.com over time, broken down by user stance.

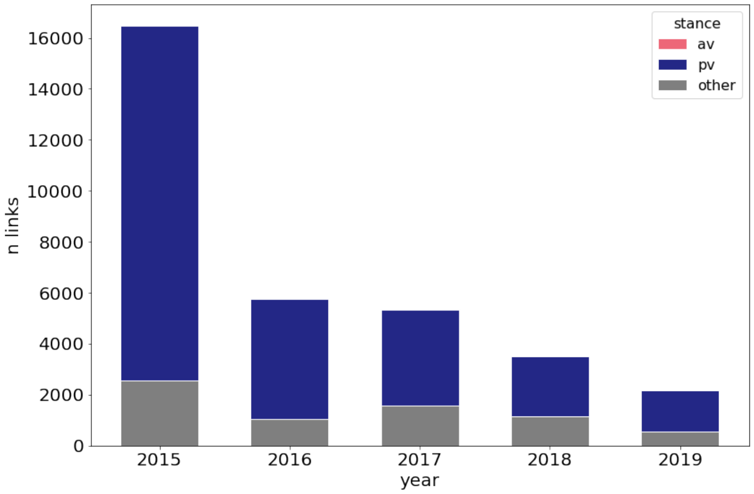

Figure 11: **bbc.co.uk links**. Illustration of how often users of different vaccination stances post links to bbc.co.uk over time, broken down by user stance.

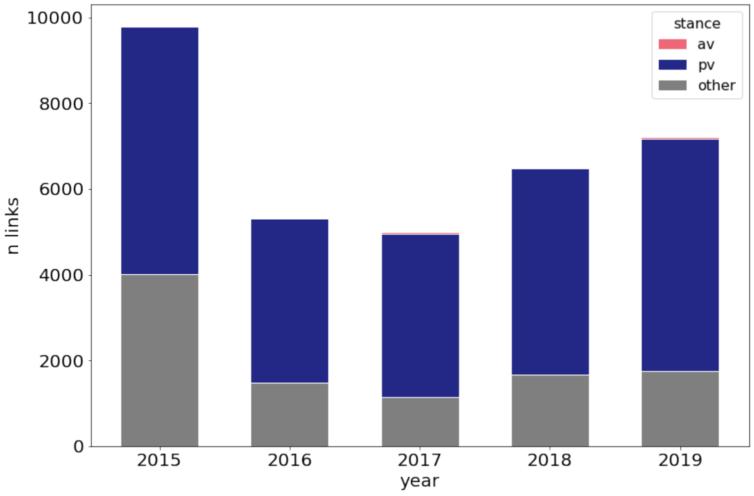

Figure 12: **nytimes.com links**. Illustration of how often users of different vaccination stances post links to nytimes.com over time, broken down by user stance.

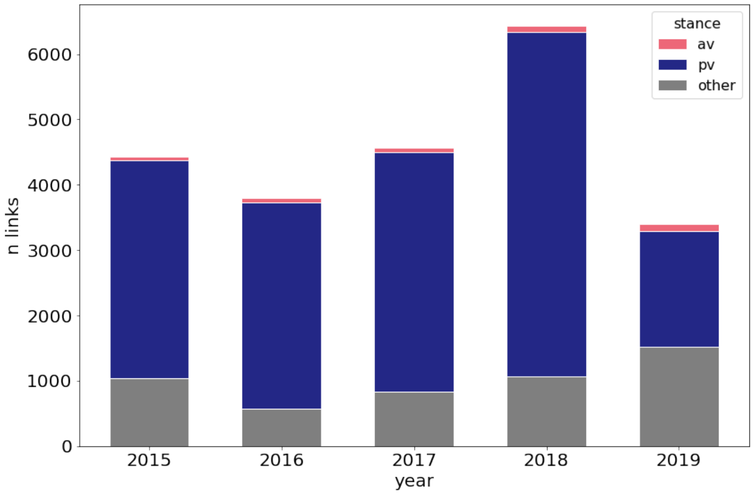

Figure 13: **cdc.gov links**. Illustration of how often users of different vaccination stances post links to cdc.gov over time, broken down by user stance.

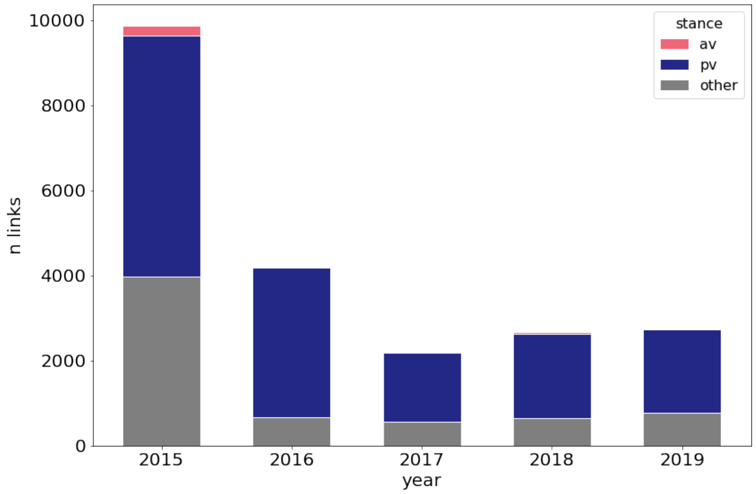

Figure 14: **edition.cnn.com links**. Illustration of how often users of different vaccination stances post links to edition.cnn.com over time, broken down by user stance.

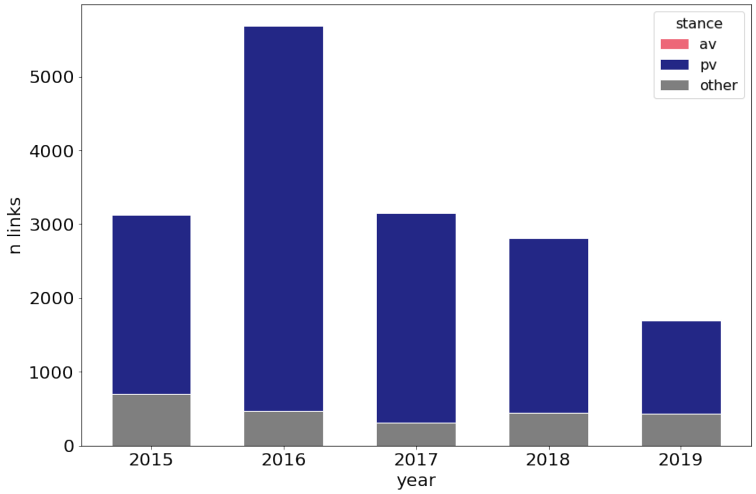

Figure 15: **reuters.com links**. Illustration of how often users of different vaccination stances post links to reuters.com over time, broken down by user stance.

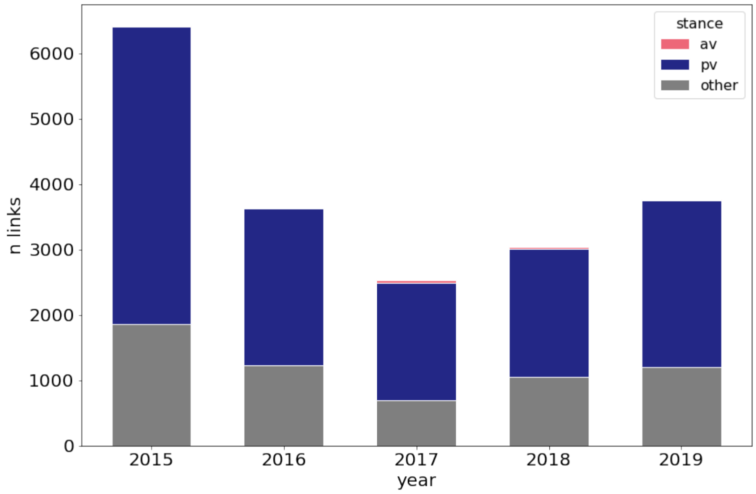

Figure 16: **theguardian.com links**. Illustration of how often users of different vaccination stances post links to theguardian.com over time, broken down by user stance.

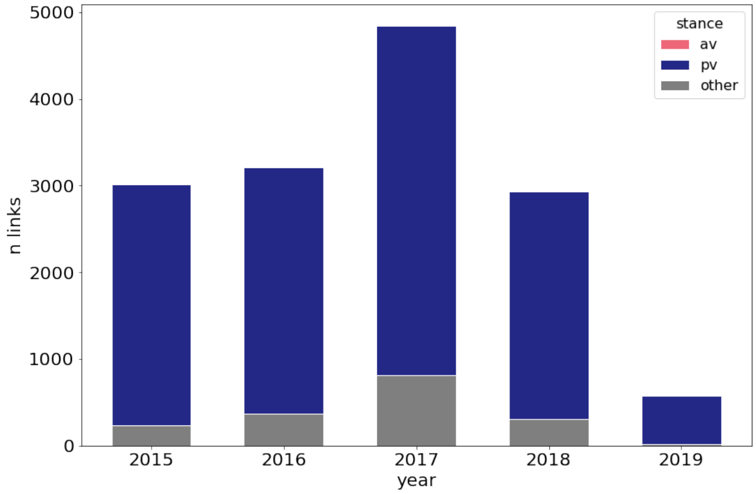

Figure 17: **bioportfolio.com links**. Illustration of how often users of different vaccination stances post links to bioportfolio.com over time, broken down by user stance.

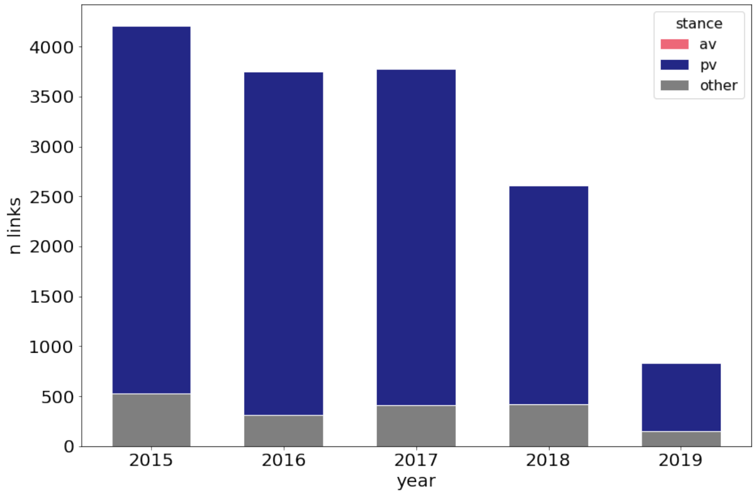

Figure 18: **sciencedaily.com links**. Illustration of how often users of different vaccination stances post links to sciencedaily.com over time, broken down by user stance.
