# Supplementary material for: Characterizing polarization in online vaccine discourse—A large-scale study
Source: PLoS One. 2022 Feb 9;17(2):e0263746. doi: 10.1371/journal.pone.0263746 (PMC8827439; doi:10.1371/journal.pone.0263746)
Supplement: S4 Appendix — contains some explorative visualizations on the interplay between user stance strength, the strength of disagreement with the user neighborhood, and the neighborhood activity and number of neighbors, for the data underlying Fig 4. (PDF) [file pone.0263746.s004.pdf]

S4 Appendix: Explorative analyses of user and neighborhood stances.

The present SI appendix contains additional analyses of the MMR graph using the stricter thresholds from the main paper.

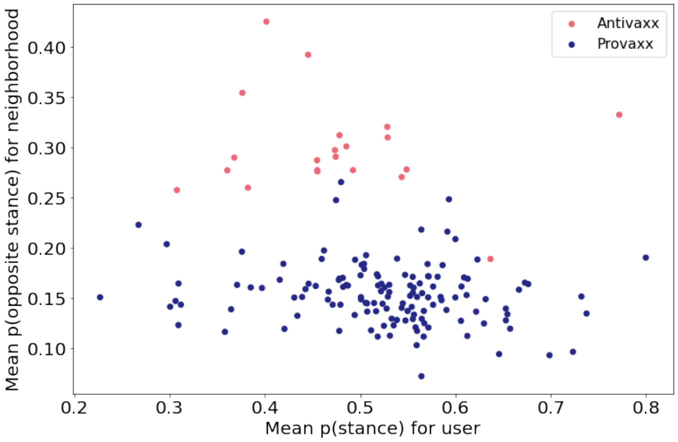

Figure 1: Comparison of user and neighborhood stances. For each user, the average tweet sentiment probability corresponding to their stance is computed. This means p_{av} for profiles with anti-vaccine stance, and p_{pv} for pro-vaccine profiles. This is depicted on the x-axis. Similarly, the average of the mean tweet probabilities for the opposite sentiment (anti-vaccine sentiment for the neighborhoods of pro-vaccine profiles and vice versa) is depicted on the y-axis. The plot only contains data points for profiles for which at least three other profiles constituted the neighborhood.

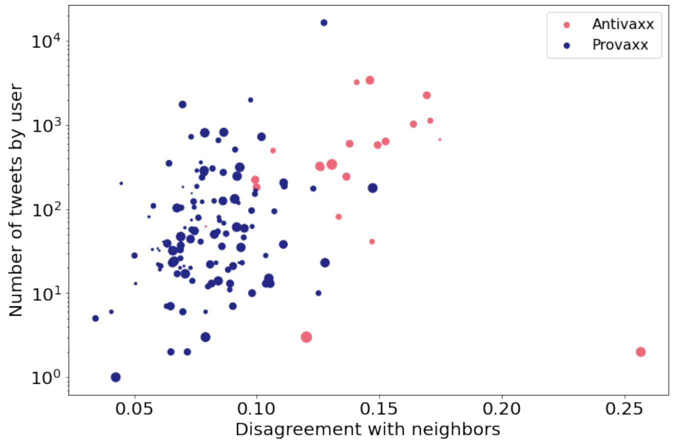

Figure 2: Similarly to Fig. 1, stances for anti-/pro-vaccine profiles and their neighborhoods are computed. These are multiplied to obtain a 'disagreement score', given by $\langle p_{\text{av, user}} \rangle \cdot \langle p_{\text{pv, neighbors}} \rangle$. Anti-vaccine profiles exhibit slightly higher disagreements with their neighbors. The sizes of each point above corresponds to the percentage of links by each user which contains links.

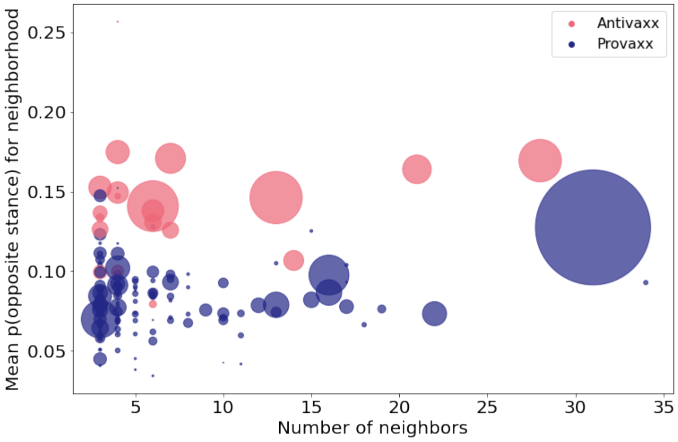

Figure 3: Illustration of profiles' disagreements with their neighborhoods by neighborhood size. Node size illustrates the number of tweets observed from each profile.
