# Supplementary material for: Characterizing polarization in online vaccine discourse—A large-scale study
Source: PLoS One. 2022 Feb 9;17(2):e0263746. doi: 10.1371/journal.pone.0263746 (PMC8827439; doi:10.1371/journal.pone.0263746)
Supplement: Attachment — Submitted filename: Review.pdf [file pone.0263746.s005.pdf]

The paper presents an interesting analysis of how Twitter behavior differs based on vaccine stances, in particular there are notable differences in the links that are shared and the degree of connection. I think the stance detection in in individual tweets is interesting methodologically and demonstrated quite rigorously. However, I think the conclusions of the analysis depend on certain assumptions made by the authors and sensitivity to those assumptions is not fully addressed. Moreover, the paper describes differences between two groups (provax and antivax accounts) but does not include uncertainty metrics for the comparisons. Finally, the authors situate their conclusions in the context of broader understandings of vaccination-related issues but not relative to other work studying discussion of vaccinations on Twitter. Acknowledging these connections would substantially improve the paper.

Major issues presented in order of importance.

1. Threshold for profile classification. Nearly all of the analysis depends on grouping accounts into provax, antivax or neutral based on a 50% threshold. While authors note that the 50% threshold is over all tweets not just vaccine-related tweets in the Materials and Methods section (to allow neutral classifications), it is necessary to note if the results are sensitive to this threshold. In particular, any account that tweets frequently about vaccines will be classified as either pro- or antivax with this procedure even if the classification model is uncertain about the actual labels. This discrete labeling from a continuous measure is a fine practice in general, but knowing if the results differ by the cutoff is necessary. In particular, smaller numbers of provax and antivax accounts will affect the uncertainty measures requested in the next point.
2. Uncertainty measures. Throughout the paper, I think the authors do not adequately report uncertainty in their measures of differences between provax and antivax accounts. In particular, the number of accounts labeled as antivax is a small subset of the full data, as shown in Figure 1. Comparing point estimates of distributions, such as in Figures 3, 4 and 7, is likely to show large differences when one group is small just due to random chance. Showing uncertainty with either confidence intervals or p-values, for example, is necessary to demonstrate that the observed differences in point estimates are not likely to have arisen due to random chance.
3. Prior work studying vaccination discourse on Twitter. Discussing prior work on the issue of vaccine debates on Twitter is necessary to present a rigorous analysis, especially to convince a reader that the authors' modeling choices are reasonable. This paper discusses prior work on the effects of antivax movements on health outcomes, but makes no mention of prior work using Twitter data to study vaccine sentiment. In particular there is a lot of research on echo chambers in vaccination debates, both COVID-19 and non-COVID-19 related. Either identifying other papers that have employed similar methods or explaining why the authors have employed different methods would improve the rigor of the analysis.
4. Correlation construction. Figure 6 shows a correlation between vaccine stance for different kinds of edges provax-provax, antivax-antivax, and provax-antivax. However, those categories are identified by that same vaccination stance. So by construction, provax-provax and antivax-antivax node pairs will be positively correlated and provax-

antivax edges will be negatively correlated. I think a global metric, such as correlation across all types of nodes, is an interesting statistic and it would be more useful here. However, using correlation between node features does require identifying an ordering for the nodes or permutations to properly measure the correlation.

Additional analyses. There are a few points in the paper that would benefit from additional analysis.

1. (Major) MMR graph (Figure 5). I think this graph is an excellent visualization of the disconnectedness of provax and antivax accounts. However, I think more should be discussed in the results.
   a. Figure 5 only shows the largest connected component. For the parts of the full graph not shown, are they mostly "pure" (all anti- or pro- vax)? Does the connectedness look different for those smaller graphs?
   b. What kind of content are the edges formed on? My reading is that the edges indicate interaction between accounts on any kind of tweet, not just vaccine-related ones. Does the anti-vax cluster interact on vaccine related tweets?
   c. Expanding on the point above, it would be nice to see if they authors can identify whether the clustering by vaccine stance is attributable to the stance or some other characteristic, i.e. are the antivax accounts densly connected *because* of their antivax stance or are they connected because they all share some other common interest and happen to be antivax. Establishing this may be challenging due to lack of reliable account-level features on Twitter data, so I understand if the authors are not able to conclusively answer this issue.
   d. There are some anti-vax accounts scattered throughout the pro-vax portion of the network and a few pro-vax accounts in the anti-vax cluster. Can you say anything about what makes those accounts (those with many connections to accounts with different stances) different from the clustered accounts? For example, are those people less anti-vax than the clustered portion? A scatterplot of each node's vaccine stance and proportion of the node's neighbors who share the same stance would get at this question.
2. (Minor) Time trends in link sharing. The dataset used to analyze link sharing (Dataset 2) spans a large time period. Do certain domains change in popularity overtime? I would think that the popularity of less-reputable sites shared by antivax accounts could ebb and flow over as their reputation changes.
3. (Minor) Geographic distribution. I do not fully see the point if including the geographic distribution of the anti-/provax tweets across US states. Is there something that explains the variation across states, maybe some demographic features of the states? I don't think this figure is necessary to include if not, especially because so few tweets are geocoded with an identifiable state.

Minor issues.

1. Why use two datasets instead of one? I think there are good reasons for why Dataset 1 is better for conducting network analysis and Dataset 2 is better for analyzing link sharing, and it would be useful for readers to explain that in the paper.

2. What inputs are used in the first layer of the tweet-level classifier? Are they word counts or some word embedding outputs?
3. In Figure 6, I do not see the need to show different curves on the same plot with different y-axes. I think the clarity of the figures would improve if split into four figures rather than two combined ones.
4. Data availability. I understand privacy concerns regarding sharing the text of tweets and account identifiers, but I do think suitably anonymized data can be shared. In particular, Dataset 1 could be shared including only the tweet-level classification scores and indicators for what base URLs were included in the tweets. Dataset 2 could be shared as just a list of interactions and account classification labels.
