# Supplementary material for: Characterizing polarization in online vaccine discourse—A large-scale study
Source: PLoS One. 2022 Feb 9;17(2):e0263746. doi: 10.1371/journal.pone.0263746 (PMC8827439; doi:10.1371/journal.pone.0263746)
Supplement: Attachment — Submitted filename: rebuttal.pdf [file pone.0263746.s006.pdf]

Dear Editor,

Thank you for the opportunity to submit a revised version of our manuscript.

We are grateful for the reviews which are very helpful, thorough, and constructive and positive. As a result of the careful comments form the reviewers, we have implemented a large number of major and minor changes in the revised text. We have worked hard to improve the paper in response to the comments, and believe the revised version presents a more coherent and clear presentation of our findings.

While detailed responses to each individual point are provided below, we wish to broadly summarize the three most significant improvements resulting from the revision process.

- We have placed a much greater emphasis on the literature on vaccine hesitancy, including recent scholarship on Twitter and other social media. The connections between our paper and the literature is also made much clearer, in several regards: In the introduction, key terminologies, themes, and results from the literature are summarized. Throughout the paper, the introduction of new concepts and framing of results and analyses is perpetually presented in the context of that summary, making both the content itself and the connection to existing scholarship clearer.
- The paper has been substantially revised featuring an entirely rewritten introduction and discussion; in the revised text, we present the main findings more cohesively and have framed them more precisely in terms of existing literature.
- A number of additional analyses have been added, including sensitivity analysis, additional uncertainty metrics, and temporal analyses of link data.

Additionally, some editorial comments requested that some information be included in the cover letter. After corresponding with a publications assistant at PLOS, the information in question is the following:

- Regarding data availability: We cannot share our full dataset publicly, as data are sensitive and not making the data public was a condition for IRB approval. The availability is updated in online form to:

  "Data cannot be shared publicly, as this was a condition for IRB approval. For inquiries regarding data access, contact compute@compute.dtu.dk."
- Regarding funding: The funding statement should be:

  "This study was funded entirely by the Danish Council for Independent Research (Project: Microdynamics of Social Interactions, grant number 4184-00556a). The funders had no role in study design, data collection and analysis, decision to publish, or preparation of the manuscript."

We hope that the editor agrees with us that the revised manuscript sufficiently addresses all concerns raised. In the following response we address editorial comments and points raised by reviewers.

Sincerely,

Bjarke Mønsted and Sune Lehmann

# Editorial comments

**Point PO.1** — Please ensure that your manuscript meets PLOS ONE's style requirements, including those for file naming. The PLOS ONE style templates can be found at https://journals.plos.org/plosone/s/file?id=wjVg/PLOSOne_formatting_sample_main_body.pdf and https://journals.plos.org/plosone/s/file?id=ba62/PLOSOne_formatting_sample_title_authors_affiliations.pdf

**Reply**: We apologize for not fulfilling all requirements the first time around. We have double checked that the revised manuscript conforms to the requirements, including file names. In the (unlikely) event that some requirements are still not met, we are happy to revise immediately.

**Point PO.2** — In your Methods section, please include additional information about your dataset and ensure that you have included a statement specifying whether the collection method complied with the terms and conditions for the website.

Furthermore, you indicated that ethical approval was not necessary for your study. We understand that the framework for ethical oversight requirements for studies of this type may differ depending on the setting and we would appreciate some further clarification regarding your research. Could you please provide further details on why your study is exempt from the need for approval and confirmation from your institutional review board or research ethics committee (e.g., in the form of a letter or email correspondence) that ethics review was not necessary for this study? Please include a copy of the correspondence as an "Other" file.

**Reply**: We have updated the data description in the methods section to explicate the fact that no terms of service agreements were violated in the data collection. Regarding ethical approval, our institution did not have a procedure for approval of analyses of online data at the time of writing the paper. However, this has since changed, and we have subsequently obtained ethical approval for the study from our IRB (IRB number COMP-IRB-2021-09). We have added a note regarding this to the materials and methods section, and will include the requested info in the revision submission form.

**Point PO.3** — Thank you for stating the following in the Acknowledgments Section of your manuscript: [The authors wish to thank Alan Mislove for his invaluable help with collection and analysis of Twitter data, and Bjarke Felbo for sharing his wisdom of machine learning. The research described in this paper was funded by the Danish Council for Independent Research, grant number 4184-00556a.] We note that you have provided funding information that is not currently declared in your Funding Statement. However, funding information should not appear in the Acknowledgments section or other areas of your manuscript. We will only publish funding information present in the Funding Statement section of the online submission form. Please remove any funding-related text from the manuscript and let us know how you would like to update your Funding Statement. Currently, your Funding Statement reads as follows:
[The authors received no specific funding for this work.]
Please include your amended statements within your cover letter; we will change the online submission form on your behalf.

**Reply**: We have removed the funding information from the acknowledgements section. After corresponding with a publications assistant at PLOS, the revised funding statement should be 'This study received funding from was the Danish Council for Independent Research (Project: Microdynamics of Social Interactions)'.

**Point PO.4** — In your Data Availability statement, you have not specified where the minimal data set underlying the results described in your manuscript can be found. PLOS defines a study's minimal data set as the underlying data used to reach the conclusions drawn in the manuscript and any additional data required to replicate the reported study findings in their entirety. All PLOS journals require that the minimal data set be made fully available. For more information about our data policy, please see http://journals.plos.org/plosone/s/data-availability.

Upon re-submitting your revised manuscript, please upload your study's minimal underlying data set as either Supporting Information files or to a stable, public repository and include the relevant URLs, DOIs, or accession numbers within your revised cover letter. For a list of acceptable repositories, please see http://journals.plos.org/plosone/s/data-availability#loc-recommended-repositories. Any potentially identifying patient information must be fully anonymized.

Important: If there are ethical or legal restrictions to sharing your data publicly, please explain these restrictions in detail. Please see our guidelines for more information on what we consider unacceptable restrictions to publicly sharing data: http://journals.plos.org/plosone/s/data-availability#loc-unacceptable-data-access-restrictions. Note that it is not acceptable for the authors to be the sole named individuals responsible for ensuring data access.

We will update your Data Availability statement to reflect the information you provide in your cover letter.

**Reply**: After corresponding with a publication assistant at PLOS, we have updated the data availability statement. The updated statement is provided in the cover letter as requested.

**Point PO.5** — We note that Figure 2 in your submission contains map images which may be copyrighted. All PLOS content is published under the Creative Commons Attribution License (CC BY 4.0), which means that the manuscript, images, and Supporting Information files will be freely available online, and any third party is permitted to access, download, copy, distribute, and use these materials in any way, even commercially, with proper attribution. For these reasons, we cannot publish previously copyrighted maps or satellite images created using proprietary data, such as Google software (Google Maps, Street View, and Earth). For more information, see our copyright guidelines: http://journals.plos.org/plosone/s/licenses-and-copyright.

We require you to either (1) present written permission from the copyright holder to publish these figures specifically under the CC BY 4.0 license, or (2) remove the figures from your submission:

a You may seek permission from the original copyright holder of Figure 2 to publish the content specifically under the CC BY 4.0 license.

  We recommend that you contact the original copyright holder with the Content Permission Form (http://journals.plos.org/plosone/s/file?id=7c09/content-permission-

form.pdf) and the following text: "I request permission for the open-access journal PLOS ONE to publish XXX under the Creative Commons Attribution License (CCAL) CC BY 4.0 (http://creativecommons.org/licenses/by/4.0/). Please be aware that this license allows unrestricted use and distribution, even commercially, by third parties. Please reply and provide explicit written permission to publish XXX under a CC BY license and complete the attached form."

Please upload the completed Content Permission Form or other proof of granted permissions as an "Other" file with your submission.

In the figure caption of the copyrighted figure, please include the following text: "Reprinted from [ref] under a CC BY license, with permission from [name of publisher], original copyright [original copyright year]."

b If you are unable to obtain permission from the original copyright holder to publish these figures under the CC BY 4.0 license or if the copyright holder's requirements are incompatible with the CC BY 4.0 license, please either i) remove the figure or ii) supply a replacement figure that complies with the CC BY 4.0 license. Please check copyright information on all replacement figures and update the figure caption with source information. If applicable, please specify in the figure caption text when a figure is similar but not identical to the original image and is therefore for illustrative purposes only. The following resources for replacing copyrighted map figures may be helpful:

- USGS National Map Viewer (public domain): http://viewer.nationalmap.gov/viewer/
- The Gateway to Astronaut Photography of Earth (public domain): http://eol.jsc.nasa.gov/sseop/clickmap/
- Maps at the CIA (public domain): https://www.cia.gov/library/publications/the-world-factbook/index.html and https://www.cia.gov/library/publications/cia-maps-publications/index.html
- NASA Earth Observatory (public domain): http://earthobservatory.nasa.gov/
- Landsat: http://landsat.visibleearth.nasa.gov/
- USGS EROS (Earth Resources Observatory and Science (EROS) Center) (public domain): http://eros.usgs.gov/#
- Natural Earth (public domain): http://www.naturalearthdata.com/

**Reply**: We apologize for the confusion. The figure in question was generated using the matplot library in Python. The map itself is drawn using methods provided by the library, which is based on an open-source framework (BaseMaps). The basemap librarys copyright terms (https://github.com/matplotlib/basemap#copyright), grants permission to use the module 'for any purpose' and does not specify any restrictions on plots generated using the framework. In the revised text, we have updated the figure caption to state that the plot was produced using an open source framework to make this point clear to the reader.

---

# Reviewer 1

We are grateful for the reviewer's helpful comments – based on which we have thoroughly revised the paper. In particular, we have deeply incorporated the vaccine hesitancy literature, and made the connections to this literature clear and explicit. Further, we have added a more thorough discussion of recent studies on social media as it pertains to vaccine hesitancy, and added some discussion of policy implications. More detailed responses to each point raised are provided below.

**Point R1.a** — The characterization of all vaccine discussions as against, for, and neutral is an oversimplification. Decades of vaccine hesitancy research has led to the understanding most individuals are not against per say, but "skeptical," referred to as vaccine hesitancy, or the "middle ground" in your conceptualization. Almost everyone has some type of hesitancy, and hesitancy depends on the vaccine and context they are in; however, the characterization here has equated hesitancy to a rejection stance, which is entirely inappropriate in my view and fully against current vaccine hesitancy discourse.

**Reply**: We agree entirely with the view expressed here of vaccine hesitancy as a continuous spectrum, and admit that a major shortcoming of the initial submission was a lack of clarity regarding the connection to vaccine hesitancy scholarship. We have revised the paper, including substantial revisions of the introduction and discussion sections, to amend this shortcoming.

We briefly summarize the changes here. First, we have included a more thorough discussion of the vaccine hesitancy literature, including research on social media, and made more explicit how our methodology pertains the terms in the literature. In particular, we have explicated our emphasis on the two 'extremes' of the hesitancy spectrum. Second, we have added additional sources and discussion regarding the connection between vaccine *hesitancy* (defined via behavior, i.e. tendency to accept/reject vaccines) and expressed attitudes regarding vaccination.

**Point R1.b** — I understand the contention for categorizing all profiles and tweets along a dichotomy of against or for vaccines. However, myself and others who know this literature well would find the findings in this paper much much more valuable if the authors were able to develop a more complex characterization that is not dichotomous. As said in the previous comment, the characterization is largely misrepresented. I would suggest authors to clearly outline how they are defining vaccine hesitancy (or related terms) using terms that are widely used by scholars in this area.

**Reply**: As mentioned above, we agree that the characterization of vaccine stance in the initial version of the paper had some significant shortcomings. The categories used in the paper and their relation to vaccine hesitancy literature are explained in much greater detail in the revised version.

In particular, we highlight the fact that while vaccine hesitancy exists on a spectrum, we focus on the two extremes on that spectrum, with the consequence that only a minority of profiles are assigned a strongly pro- or anti-vaccine stance. We have also made more clear the fact that later analyses, such as those visualized in Figs 6 and 7 (5 and 6 in the revised version), do in fact view user vaccine stances as a continuous measure, given by averages over the sentiment expressed in individual tweets by the user.

Throughout the revised manuscript (particularly in the discussion), we have updated the text to much more clearly relate to the terminology used in vaccine hesitancy literature.

**Point R1.c** — I would have liked to see a much more elaborate exploration of the implications of your findings for the literature and policymaking. I get the impression that the wealth of literature on vaccine hesitancy is largely unexplored or ignored. However, this may not be completely accurate. For example, commercial interests have actually been explored in literature in the past few years. These interests are primarily from the focal point of trust, whereas mistrust is directed towards government, providers, and pharma, to direct trust towards companies or groups that do not fall in the canonical communities. These issues have been explored and should be mentioned in your discussion. I would suggest a more comprehensive searching of the literature, including recently published reviews on the topic. Furthermore recent work on COVID-19 vaccine hesitancy have also explored some of these topics, and they are relevant for today's discussions.

**Reply**: As for the hesitancy literature, we agree with the point raised here. In addition to the changes outlined in the response to the previous comments, we have rewritten the introduction to summarize from the literature the most ubiquitous factors concerning vaccine hesitancy, and summarized recent findings on how each factor is expressed in the context of social media.

In terms of the excellent remark on policy implications, we have added new considerations about possible implications, as well as suggestionsfor possible future avenues of research.

---

## Reviewer 2

We are thankful for the positive remarks and helpful comments raised here. The main changes brought about by the points raised here are (1) a much more rigorous analyses of robustness to stance labelling, and (2) a rewrite of large parts of the paper, especially the introduction and discussion parts, to make the story more cohesive.

**Point R2.a** — Perhaps this is just my taste, but my main suggestion for the authors is to do more to situate their very impressive data collection and methodology and really drive home what they think we've learned from all of this. The findings are each individually interesting and do stand on their own, but I think they could be tied together into a more cohesive story. To me, there are at least two fascinating findings here that quantify dynamics that have previously been identified qualitatively — and are each more interesting than the "polarization" referenced in the title (after all, why wouldn't we expect vaccine discourse to polarize in a venue like Twitter, where people tend to express stances that are more strongly held?).

First, the grift. Journalistic accounts have documented how various conspiracy theorists on the internet try to channel their followers' fears into product sales, but this is the first time I have seen it quantified. Second, and more importantly, the interaction network. So much work on vaccine misinformation is about exposure to various sources that are flagged as unreliable or misleading — with the implication that if we were better at flagging or removing bad information from these sites, people's attitudes and behaviors would change in pro-social ways. This work

treats Twitter as social *media.* What I take from these findings is that this is not even half of the story — the real action is in the communities that get built on these sites. Which is to say, the really compelling part of this manuscript is its treatment of Twitter as a social *network.* I suspect that no amount of flagging unreliable domains will deter users in these communities from reinforcing each others' anti-vaccine views — Motta, et al (2021) on anti-vaccine social identities (https://www.tandfonline.com/doi/full/10.1080/21565503.2021.1932528?journalCode=rpgi20) could be a helpful tie-in on this front.

This presents some interesting and tough questions concerning the tradeoffs associated with so much information flowing in these online spaces. Here, Twitter is doing exactly what it's designed to do: bring people together to share and discuss information. It's agnostic as to the ends for which it is bringing people together, and it's legitimately difficult to say with any certainty what we should expect them to do regarding *communities of users* who reinforce each others' anti-vaccine views. That's a harder problem than picking some unreliable information sources to flag or even block.

**Reply**: These are very good points, and we agree that the overall 'story' of the first version of our text could be much more clear and concise. We genuinely appreciate the reviewer's clearheaded analysis of the implications of our work. In response, we have edited the revised version to make the main messages more clear.

In particular, we have highlighted already in the introduction the main themes proposed in this point, and made clear how they pertain to the vaccine hesitancy literature. The discussion then returns to these themes and emphasizes the findings regarding commercial conflicts of interest, as well as the difficulties which tightly knitted communities holding fringe views present, and the necessity of better understanding the interplay between network structure, attitude, and information sources, in the context of online misinformation.

**Point R2.b** — How sensitive are these results to higher thresholds for classifying users' stances? 50% of on-topic tweets with at least 50% p(stance) is an intuitive threshold, but my expectation would be that most tweets with a stance express an unambiguous stance — i.e. p(stance) well over .5 — and most users with a stance are much more consistent than 51%. How much data do we lose when we require more confidence in our classifications?

**Reply**: We agree that the effect on our results of increasing the sentiment threshold should be investigated. In response to this point, and to Point R3.b which expresses similar concerns, we have added a supplementary information appendix, SI1, to address this point.

To summarize, the findings are generally not substantially changed. The one exception to this, is that when placing extremely strict demands for vaccine stands, the 'news' category becomes increasingly associated with anti-vaccine stances, and decreasingly associated with pro-vaccine stances. This turns out to be due to Fox News being an extremely popular source among users expressing the strongest anti-vaccine sentiments.

Regarding the matter of data loss as the threshold is modified: The data loss is gradual and quite significant, with approximately 90% of profiles not assigned either label as the sentiment threshold is increased from 50% to 90%. This is illustrated in the section on data retention in SI1. We hope that this adequately addresses the concerns expressed in this point.

**Point R2.c** — Does downsampling instead of upsampling matter? There's a ton of data here and also a ton of imbalance, so I worry that the relatively smaller number of anti-vax tweets wind up getting overworked.

**Reply**: It is true that upsampling can result in overfitting, in the sense that the model will be overly dependent on patterns found in the upsampled data points. However, we are certain that this is not the case here, as overfitting would cause decreased performance in the validation set when running cross-validation. Using regularization techniques like the dropout layers shown in the ML architecture further allows the training procedure to rectify this. It is for this reason upsampling is a standard procedure in related literature, such as the DeepMoji paper, which our architecture is based upon. We have updated the paper to explicate that the upsampling is strictly done within each cross-validation training dataset, and added a reference to the DeepMoji paper.

**Point R2.d** — Personally, the geolocation and state-level analysis didn't do much for me. The share of tweets/users who could be geolocated in the first place is so small, and those users are likely different from users in similar locations who can't be located. I wouldn't put my foot down and say it's wrong or needs to be cut, but I would say that I wouldn't miss it (it doesn't even get a full paragraph in the body as it stands). I'm just not sure what it adds.

**Reply**: It's true that very few tweets can be *directly* geolocated. However our large dataset (Dataset 1) puts us in a unique position to infer the location of many of the tweets which are not encoded with GPS coordinates. We agree, however, that the figure is not at all central to the paper's argument. It may be relevant to some readers interested in e.g. effects of US state policies on Twitter discourse. Therefore, we have moved it to the supporting information. A similar point is raised in Point R3.h, where we provide additional details regarding this decision, which we hope addresses the reviewer's concern.

---

## Reviewer 3

We are very grateful for the thorough review and have made a number of changes to the paper in response to the points raised. To summarize in broad terms the changes made in response to the points raised: First, a number of additional analyses have been added concerning the temporal dynamics of information sources, and the interplay between the strength of stances for users and their neighbours in the interaction graph. Secondly we have added new analyses regarding uncertainties and robustness, including a more thorough discussion of related research and how it related to the findings. A detailed response to each individual point is provided below.

**Point R3.a** — The paper presents an interesting analysis of how Twitter behavior differs based on vaccine stances, in particular there are notable differences in the links that are shared and the degree of connection. I think the stance detection in in individual tweets is interesting methodologically and demonstrated quite rigorously. However, I think the conclusions of the analysis depend on certain assumptions made by the authors and sensitivity to those assumptions is not fully addressed. Moreover, the paper describes differences between two groups (provax and anti-vax accounts) but does not include uncertainty metrics for the comparisons. Finally, the authors

situate their conclusions in the context of broader understandings of vaccination-related issues but not relative to other work studying discussion of vaccinations on Twitter. Acknowledging these connections would substantially improve the paper.

**Reply**: We agree that the problem of sensitivity to assumptions, specifically the sentiment threshold for assigning vaccination stances at the user level was a weakness of our initial submission. In response, we have added an SI appendix with robustness analysis probing the effects of altering the threshold, which turned out to reveal a new interesting finding regarding the relation between news sharing and the strength of anti/pro-vaccine stances. We provide additional more detail regarding this in the response to Point R3.b.

In addition, we have added uncertainty metrics have been added to all figures, and the methods for computing errors is more thoroughly discussed in the revised paper. More details are provided in the response to Point R3.c.

Finally, we have added a summary of recent research on vaccine hesitancy/discourse on social media to the introduction, and refer back to this throughout the analyses and discussion to make the connections more clear to the reader. More detail is provided in the response to Point R3.d.

## Major

**Point R3.b** — Threshold for profile classification. Nearly all of the analysis depends on grouping accounts into provax, antivax or neutral based on a 50% threshold. While authors note that the 50% threshold is over all tweets not just vaccine-related tweets in the Materials and Methods section (to allow neutral classifications), it is necessary to note if the results are sensitive to this threshold. In particular, any account that tweets frequently about vaccines will be classified as either pro- or antivax with this procedure even if the classification model is uncertain about the actual labels. This discrete labeling from a continuous measure is a fine practice in general, but knowing if the results differ by the cutoff is necessary. In particular, smaller numbers of provax and antivax accounts will affect the uncertainty measures requested in the next point.

**Reply**: This is a good point. Point R2.b expresses similar concerns, and we have to agree completely that missing robustness analyses was a major weakness of the first version of the paper. We have now added a supplementary information appendix (S1) to the revised manuscript, where we investigate the effects of increasing the sentiment threshold such that profiles must express stronger sentiments regarding vaccines to fall in either category, on the results.

Increasing this threshold does not generally change the results, with one notable exception: As the threshold is increased to 90%, AV links are *more* likely than PV to share links in the 'news' category - opposite of the finding in the main paper. Investigating the link distribution reveals that this is due to Fox News being a highly popular source among extremely anti-vaccine profiles.

This is an interesting additional finding, and we are grateful for this comment for prompting it.

Further, we would like to clarify that tweet sentiments are *not* calculated for all tweets, but only for tweets containing vaccine-related keywords. Tweets are classified as neutral/unrelated if they express sentiment that is not overly positive or negative regarding vaccination, or unrelated to human vaccination

(such as mentioning vaccination of pets, etc.). For this reason, accounts that frequently tweet about vaccines will not generally be assigned to either (anti/pro) category, unless they consistently express strong positive or negative sentiments on the subject. We have updated the materials and methods section to make this point more clear.

Finally, we add that not all of our analyses depend on partitioning users into discrete groups. The analysis shown in figure 6 (now figure 5), for instance, does not look at discrete stances but instead considers only the average probabilities of anti/pro-vaccine sentiments assigned to each user's tweets by our classifier, yet the finding remains that user stances are positively correlated with that of their neighbors.

**Point R3.c** — Uncertainty measures. Throughout the paper, I think the authors do not adequately report uncertainty in their measures of differences between provax and antivax accounts. In particular, the number of accounts labeled as antivax is a small subset of the full data, as shown in Figure 1. Comparing point estimates of distributions, such as in Figures 3, 4 and 7, is likely to show large differences when one group is small just due to random chance. Showing uncertainty with either confidence intervals or p-values, for example, is necessary to demonstrate that the observed differences in point estimates are not likely to have arisen due to random chance.

**Reply**: We fully agree that care must be taken to disentangle true differences in the underlying distributions from differences arising from noise, especially when comparing small datasets. We think there are two additional considerations to take into account:

a The dataset is very large, and the smallest group of profiles (anti-vaccine) comprises over 40,000 accounts, and errorbars on some of the figures are so small that they are difficult to see due to being smaller than the linewidth.

b As noted above in Point R3.b, results can also be sensitive to parameter choices, in particular the tweet sentiment threshold. Small changes that result in changes much larger than the error bars.

For these reasons, we believe it is appropriate to discuss uncertainties and robustness simultaneously in cases where the error bars are extremely small.

We do this in the first section of the newly added SI1, where we also include data tables containing the exact values for figure data and error bars, in cases where the latter are too small to be depicted visually. Further, section 2 of SI1 repeats the figures in question when increasing the tweet sentiment threshold. This occasionally results in large the error bars, because the stricter criteria for stance association reduces the number of profiles labeled AV/PV. The amount of data retained for each such sentiment threshold is summarized in section 3 of SI1. In addition to the inclusion of SI1, some additional changes have been made to the paper in response to this point:

- Error bars have been added to figure 6 (formerly figure 7) - the Jensen-Shannon distance plot - using a bootstrap estimation procedure.
- Details have been added to the caption of figure 6 (formerly figure 5) to explain how the error bars on Pearson correlations are calculated.

- A note on uncertainties and robustness checks wrt. the thoughts expressed here has been added to the materials and methods section.

**Point R3.d** — Prior work studying vaccination discourse on Twitter. Discussing prior work on the issue of vaccine debates on Twitter is necessary to present a rigorous analysis, especially to convince a reader that the authors' modeling choices are reasonable. This paper discusses prior work on the effects of antivax movements on health outcomes, but makes no mention of prior work using Twitter data to study vaccine sentiment. In particular there is a lot of research on echo chambers in vaccination debates, both COVID-19 and non-COVID-19 related. Either identifying other papers that have employed similar methods or explaining why the authors have employed different methods would improve the rigor of the analysis.

**Reply**: We agree that the involvement of literature in the first version of the paper was insufficient. To rectify this issue, we have implemented a number major changes to the paper:

- The revised version of the paper connects more deeply and explicitly to recent scholarship on the topic of vaccination discourse and vaccine hesitancy in the context of social media.
- The literature on vaccine hesitancy in general is now included to a much larger degree, and the findings and areas of focus from the aforementioned research on Twitter/social media discourse is related explicitly to the hesitancy literature.
- Finally, the findings and analyses in the paper are more explicitly being related to the findings and terminologies of existing literature.

Some similar concerns were voiced earlier, in Points R1.a to R1.c. The responses there contain additional details regarding the connections between vaccine hesitancy literature (social media and generally), and the terminology and findings of the paper.

**Point R3.e** — Correlation construction. Figure 6 shows a correlation between vaccine stance for different kinds of edges provax-provax, antivax-antivax, and provax-antivax. However, those categories are identified by that same vaccination stance. So by construction, provax-provax and antivax-antivax node pairs will be positively correlated and provax-antivax edges will be negatively correlated. I think a global metric, such as correlation across all types of nodes, is an interesting statistic and it would be more useful here. However, using correlation between node features does require identifying an ordering for the nodes or permutations to properly measure the correlation.

**Reply**: It is absolutely true that a global metric is superior in this case. We have not been sufficiently clear regarding what the correlations in question represent, and have updated the caption of the figure and the surrounding text to make this more clear.

To briefly reiterate exactly how figure 6 (now figure 5) is constructed:

1. We consider all users with sufficiently many tweets about vaccination.

2. For all tweets for each user, a $p_{\text{av}}$ and $p_{\text{pv}}$ are computed, and the average $p_{\text{av}}$ and $p_{\text{pv}}$ for each user is calculated.

3. Considering all pairs of nodes $(u, v)$ in the interaction network, we then compute the correlation between e.g. $p_{\text{av}}$ for nodes and neighbors.

As also described in the response to Point R3.b, the aim is precisely to probe what happens when user stances are not discretely partitioned into av/pv, but instead viewed as a continuum based on the mean vaccine sentiment expressed in tweets. The resulting correlations are not due to the previously applied av/pv labels, but depend only on these mean sentiments, and the structure of the interaction network. To highlight this, we have reconstructed the figure, using a random permutation of nodes, resulting in the correlations disappearing, as shown in Fig. 1. We have updated the figure caption, as well is the

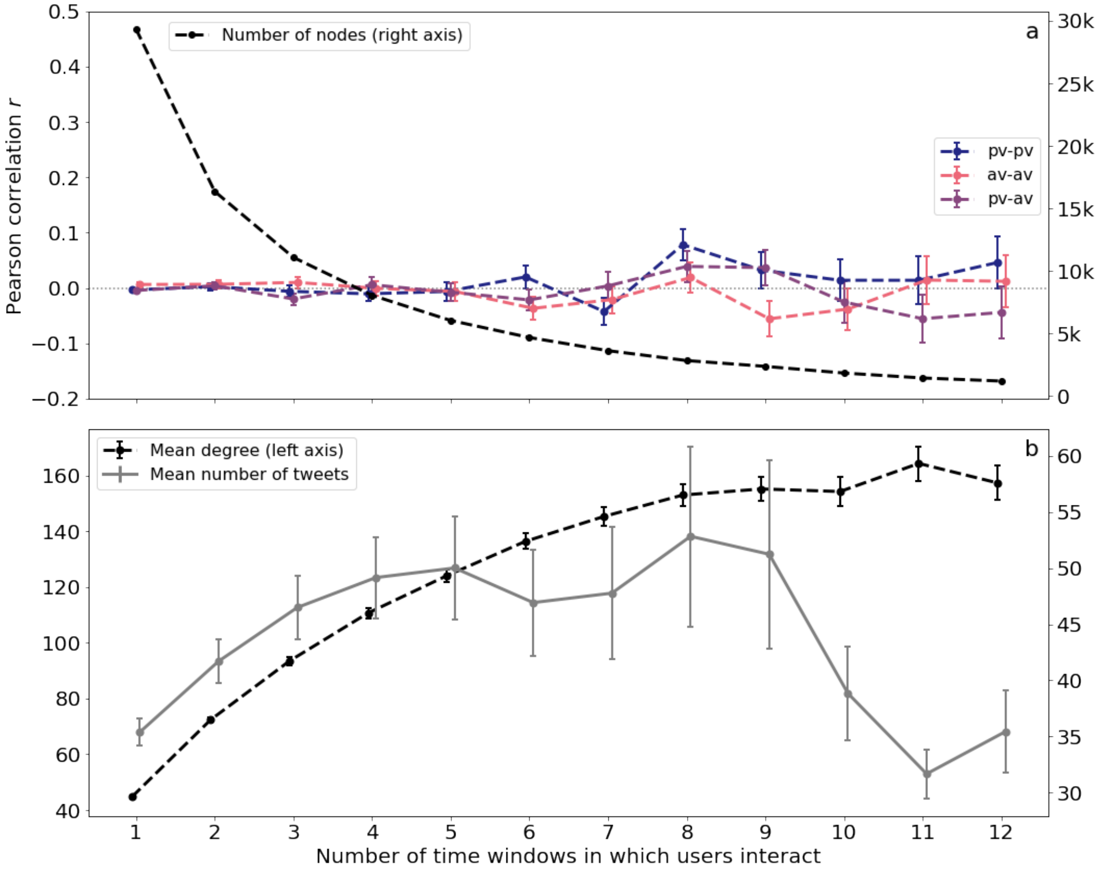


Figure 1: Illustration of the correlations between P(av) and P(pv), the mean estimated probabilities of users' tweets expressing negative and positive sentiments, respectively, regarding vaccines. To verify that the correlations depicted in the paper are in fact due to network structure, we have randomly permuted the nodes in the interaction graph and recalculated the correlations, which are now close to zero.

corresponding description of the underlying analysis in the revised text, to make it more clear that this analysis indeed considers the overall correlations between average sentiment, rather than the discrete

labels. Finally, the legends in figure 6 (now figure 5) have been updated to make more clear that the quantity depicted is the correlation between probabilities.

## Additional analyses

**Point R3.f** — (Major) MMR graph (Figure 5). I think this graph is an excellent visualization of the disconnectedness of provax and antivax accounts. However, I think more should be discussed in the results.

a Figure 5 only shows the largest connected component. For the parts of the full graph not shown, are they mostly "pure" (all anti- or pro- vax)? Does the connectedness look different for those smaller graphs?

b What kind of content are the edges formed on? My reading is that the edges indicate interaction between accounts on any kind of tweet, not just vaccine-related ones. Does the anti-vax cluster interact on vaccine related tweets?

c Expanding on the point above, it would be nice to see if they authors can identify whether the clustering by vaccine stance is attributable to the stance or some other characteristic, i.e. are the antivax accounts densly connected because of their antivax stance or are they connected because they all share some other common interest and happen to be antivax. Establishing this may be challenging due to lack of reliable account-level features on Twitter data, so I understand if the authors are not able to conclusively answer this issue.

d There are some anti-vax accounts scattered throughout the pro-vax portion of the network and a few pro-vax accounts in the anti-vax cluster. Can you say anything about what makes those accounts (those with many connections to accounts with different stances) different from the clustered accounts? For example, are those people less anti-vax than the clustered portion? A scatterplot of each node's vaccine stance and proportion of the node's neighbors who share the same stance would get at this question.

**Reply**: We respond to the comment above above point by point below.

a The figure in question depicts the MMR graph constructed using very strict criteria for link and node inclusion. Specifically, for node inclusion, we demand that nodes express consistently strong vaccine sentiments in several time windows, and for link inclusion that nodes interact mutually in several such time windows. The result is a graph in which fairly few nodes are retained, and which one may think of as representing the most 'involved' participants in the vaccine discourse. The subsequent analyses aim to investigate the effects of making the aforementioned criteria less strict.

  We note first of all that we do not believe we have expressed this sufficiently clearly in the paper, and have remedied this by revising the beginning of section 2.

  Regarding the remaining components in figure 5 (now figure 4), all are very small (containing fewer than 30 nodes in each), and contain relatively fewer anti-vaccine-profiles (5.6%). A more

detailed analysis of such very small clusters does not seem to promise any deeper insights, though, so we stick to visualizing the giant component initially, as the subsequent analyses focus on the entire graph. We have added some details including these numbers to the description of the version of the MMR graph illustrated in figure 5 (now figure 4).

b The edges are indeed built using Dataset 1, i.e. the large dataset not specific to vaccines. However, only nodes corresponding to users who tweet content with vaccine-related keywords are retained. The edges are formed by connecting users $u$ and $v$ if and only if user $u$ retweets or mentions user $v$, and user $v$ retweets or mentions user $u$ within the same 3-month time window, unless otherwise specified - figure 6 (now figure 5) for instance, illustrates the effects of demanding that reciprocal interaction must take place in several such time windows. As also noted in the response to Point R3.i, building the interaction network is a quite heavy technical task, and running subsequent text analyses on top of that would only add to the challenges. Further investigating the text content, not just in anti-vaccine clusters but at the community level in general, would be interesting for a followup study, and we have noted this idea as a suggestion for future research in the paper.

c As suspected, it would be very challenging to say anything remotely conclusively regarding the underlying cause for the formation of the clusters. However, there has been some work in recent years proposing and empirically/numerically evaluating various mechanisms for such clusterings. We have added at the end of section 2 some discussion of such mechanisms which are consistent with our findings.

d This is a great question. We conducted some additional explorative analyses to investigate this, but cannot identify any strong determinants of this, only that anti-vaccine profiles generally tend to disagree more with their neighborhood independent of how 'strong' their stance is, and that AV profiles are more active across the board. We have added a brief discussion of this in the main paper, and included details and figures in SI appendix 4.

**Point R3.g** — (Minor) Time trends in link sharing. The dataset used to analyze link sharing (Dataset 2) spans a large time period. Do certain domains change in popularity overtime? I would think that the popularity of less-reputable sites shared by antivax accounts could ebb and flow over as their reputation changes.

**Reply**: The answer seems to be that some links change quite a bit in popularity, in particular the more conspiracy-related ones. However, the relative popularity between the different user stances appears quite constant. We have added a bit of discussion of this in the section on link analysis, and added detailed plots for each target URL in appendix S3.

**Point R3.h** — (Minor) Geographic distribution. I do not fully see the point if including the geographic distribution of the anti-/provax tweets across US states. Is there something that explains the variation across states, maybe some demographic features of the states? I don't think this figure is necessary to include if not, especially because so few tweets are geocoded with an identifiable state.

**Reply**: This is an excellent point, and was also raised in Point R2.d. The idea behind including the figure in the first place was that an ongoing area of research is the effects of policy on e.g. vaccination rates. US states are often used in such research, as vaccination-related policies often differ between the various states. However, we have to agree that this is only somewhat peripherally related to the present paper, and should not be included in the main text. We are disinclined, however, to removing entirely, the reasons being that a) researchers of the aforementioned kind might find the results interesting, and b) our large dataset (Dataset 1) allowed us to infer the location at the state level for a relatively large number of tweets, whereas other researchers might be limited by the very low rate of GPS-encoded tweets, as also noted by the reviewer. We have therefore moved the figure, along with the corresponding descriptions of methodology for geolocation, to a SI appendix which also contains the raw underlying data, in the hope that they be of use to other researchers. We hope that this adequately addresses the concern raised.

## Minor issues

**Point R3.i** — Why use two datasets instead of one? I think there are good reasons for why Dataset 1 is better for conducting network analysis and Dataset 2 is better for analyzing link sharing, and it would be useful for readers to explain that in the paper.

**Reply**: The decision to explain separately the two subsets of the data referred to as Datasets 1 and 2 was made in an attempt to clarify to the reader that some parts of the data were obtained in different manners, and, as noted by the reviewer, suited for different analyses. Specifically analyzing overall interactions using Dataset 1, due to its size, and analyzing vaccine discourse using Dataset 2, due to it being more particular to vaccine discussions because it was obtained querying for vaccine-related terms.

By separating the two descriptions, we hope to avoid unnecessary confusion as to which data is used in which analyses. Additionally, constructing the interaction graph from Dataset 1 presented some quite significant technical challenges. The partitioning into two distinct datasets also serves to reflect the scoping of the problem in the sense that we opted to first solve the problem of constructing the interaction graph, then use that as a stratum for investigating interaction network structure in relation to vaccine sentiments and stances. Based on this reviewer point, we have added a short paragraph clarifying this issue, in the section on twitter data under 'Materials and methods'.

**Point R3.j** — What inputs are used in the first layer of the tweet-level classifier? Are they word counts or some word embedding outputs?

**Reply**: The first (input) layer contains one-hot encoded representations of individual words. This is similar to the two models our architecture builds upon (the DeepMoji sentiment classifier and the FastText classifier). The weights from there to the subsequent embedding layers are fitted during the training procedure. We have updated the figure description to be more clear regarding the input layer, as well as the distinction between the DeepMoji and FastText classifiers.

**Point R3.k** — In Figure 6, I do not see the need to show different curves on the same plot with different y-axes. I think the clarity of the figures would improve if split into four figures rather than two combined ones.

**Reply**: We do agree that using dual y-axes tends to reduce clarity in terms of visually processing each individual line. Dual y-axes should not be used merely to 'squeeze' more data into a single plot. However, the tradeoff tends to be that interactions between each line become harder to visually process when the reader has to switch between several graphs. This tradeoff tends to take a central role when discussing whether dual axes are appropriate, cf. for instance the following post on data visualization and dual axes (`https://datahero.com/blog/2015/04/23/the-dos-and-donts-of-dual-axis-charts/`). The ambition underlying the figure in question is to help the reader understand what happens when stricter contact requirements are enforced before connecting two nodes in the contact graph. The key quantities of interest here are not so much the direct effects of this (such as the number of nodes decreasing as requirements grow more strict), as the interactions between these. For this reason, we feel that the use of dual axes, in this particular case, is justified.

**Point R3.1** — Data availability. I understand privacy concerns regarding sharing the text of tweets and account identifiers, but I do think suitably anonymized data can be shared. In particular, Dataset 1 could be shared including only the tweet-level classification scores and indicators for what base URLs were included in the tweets. Dataset 2 could be shared as just a list of interactions and account classification labels.

**Reply**: We agree that sharing anonymized data is generally a great practice. However, in the context of Twitter data, and large data sets in general, properly anonymization becomes quite difficult, due to the possibility of combinations of features uniquely identifying users. For instance, sharing a number of tweet probabilities along with a list of links shared might uniquely identify users. Network data in particular are especially vulnerable to de-anonymization[1]. Finally, making only aggregate results publicly available was a criterion for IRB approval. For these reasons, we do not think it possibly, unfortunately, to make the data publicly available.

However, as also mentioned in Point PO.4, we will provide a point of contact at our institution were researchers interested in using the data can request access, and we will update the data availability statement accordingly.

# References

[1] Romanini D, Lehmann S, Kivelä M. Privacy and uniqueness of neighborhoods in social networks. Scientific reports. 2021;11(1):1–15.
